# Shaping Properties and Outcomes of Nickel-Titanium Reciprocation Systems in Primary Teeth: A Systematic Review and Meta-Analysis of In Vitro Studies

**DOI:** 10.7759/cureus.30995

**Published:** 2022-11-01

**Authors:** SelvaKumar Haridoss, Bhavyaa R, Kavitha Swaminathan, Aruna P

**Affiliations:** 1 Pedodontics and Preventive Dentistry, Sri Ramachandra Institute of Higher Education and Research, Chennai, IND; 2 Pediatric and Preventive Dentistry, Sri Ramachandra Institute of Higher Education and Research, Chennai, IND

**Keywords:** systematic review and meta analysis, pediatric endodontics, root canal therapy, primary teeth, rotary instruments, reciprocating instruments

## Abstract

The aim of this systematic review was to investigate the shaping properties and outcomes of reciprocating instruments in primary teeth. A search was conducted across various electronic databases such as PubMed, Scopus, EBSCO (dentistry and oral health sciences), LILACS, Cochrane, Google Scholar (first 100 hits), Open Grey, Ovid, and Shodhganga. Two authors independently performed data extraction and quality assessment of the selected articles using Rayyan software. A total of 12 studies were included. All 12 included studies qualified for qualitative analysis and five for meta-analysis. The meta-analysis revealed there was no statistical significance between rotary and reciprocating instruments concerning instrumentation time and canal transportation. The rotary and reciprocating instruments showed better shaping outcomes than hand instruments. Randomized controlled trials are required with adequate quality to perform a meta-analysis to provide better and more substantial evidence to use reciprocating instruments.

## Introduction and background

The main objective of endodontic treatments is to sustain the integrity and function of teeth and periradicular tissues [1]. Conventional endodontic treatment using hand instruments for cleaning and shaping primary teeth is time‑consuming and often causes fatigue to the operator and child [2]. In 2000, Barr et al. introduced rotary endodontics in pediatric dentistry [3]. Rotary nickel-titanium (NiTi) files follow the original anatomy of curved canals in primary teeth and minimize the risk of procedural errors [4]. Additionally, funnel-shaped canal preparation can be obtained using rotary instruments, thereby enabling us to achieve a uniform and more predictable obturation [3]. The advantage of using rotary files is that they reduce preparation time considerably and, as a result, increase the child’s cooperation in the endodontic procedure [5].

Rotary files maintain longitudinal root canal geometries [6]; however, most of these systems recommend the use of a series of files to accomplish the final shape [7]. Moreover, the separation of the files is a major drawback of rotary NiTi instruments [8]. Yared (2008) proposed a new type of file called reciprocating files that use reciprocating motions [9,10]. Reciprocating files are made of an M-wire NiTi alloy and mounted on a dedicated handpiece and motor to operate the reciprocating rotation [11,12]. The counterclockwise rotation cuts dentin, and the reversing clockwise movement releases the file from the canal wall [13]. Moreover, the characteristic design of blades permits the operator to apply low instrumentation force coupled with a low risk of iatrogenic errors [14,15].

A literature search on the comparison of the shaping ability of different file systems shows that two-dimensional (2D) radiographs or serial sectional methods are commonly used in research, while studies with computed tomography (CT) have also been documented. The shaping properties and outcomes of rotary and reciprocating instruments in permanent teeth have been studied extensively. However, because primary teeth possess bizarre root canal anatomy, assessing the shaping ability of different instruments is critical. The introduction of reciprocation instruments in primary teeth is relatively recent and numerous studies have been performed to evaluate its efficacy. To our knowledge, no systematic review has been documented regarding the same. Hence, the aim of this systematic review was to investigate the shaping properties and outcomes of reciprocating and rotary instruments in primary teeth.

## Review

Methodology

Protocol and Registration

This systematic review was done according to Preferred Reporting Items for Systematic Reviews and Meta-Analyses (PRISMA) guidelines and was registered in the PROSPERO (Centre for Reviews and Dissemination, University of York; http://www.crd.york. ac.uk/PROSPERO) with registration number CRD 42022315465.

Research Question

A research question was devised based on the PICOS format proposed by Page et al. [16]. In extracted primary teeth (P), does the use of reciprocating instruments (I) confer better shaping ability (O) in in-vitro studies (S) compared to rotary and hand instruments?

Inclusion Criteria

We included studies investigating one or more shaping properties and outcomes of reciprocation instruments compared to rotary or hand instruments, in-vitro studies using extracted primary teeth, and studies published in the English language

We excluded literature reviews, opinion articles, letters, case series, case reports, conference abstracts, and those which involved artificial teeth. Further, studies that were conducted using hand instruments in replicating the reciprocating motion without the usage of reciprocating instruments were excluded.

Search Strategy and Study Identification

The key search terms used were “Reciprocation instruments,” “Rotary NiTi instruments,” “Endodontic treatment,” and “In vitro studies” modified in terms of the glossary of each database and combined using Boolean operators. The search was carried out by one of the reviewers (SKH) for potential studies in databases such as PubMed, Scopus, EBSCO (dentistry and oral health sciences), LILACS, Cochrane, Google Scholar (first 100 hits), Open Grey, Ovid, and Shodhganga from January 2000 to June 2021. A literature search was conducted starting from the year 2000 since Barr et al. [3] was the first article published on the use of rotary instruments in primary teeth. The references from the included studies, published reviews, and standard pediatric textbooks were screened. In addition, hand searches were conducted in pediatric dentistry journals, the International Journal of Pediatric Dentistry, the Journal of Indian Society of Pedodontics and Preventive Dentistry, and the Journal of Clinical Pediatric Dentistry. Zotero software was used to remove any duplicates and select eligible studies from the database findings and other sources (lists of references in included studies). Two independent reviewers (SKH and BR) with experience in conducting systematic reviews selected eligible studies using the web-based Rayyan software developed by Ouzzani et al. [17]. Any disagreement between reviewers was resolved by a third reviewer (KS).

Data Extraction

The two reviewers (SKH and BR) extracted data systematically from the included studies. A standardized, pre-piloted form was used to enter the data. The corresponding author was personally contacted through the mail to retrieve data in case of missing or unclear data. The third reviewer (KS) was consulted to solve any discordance/disagreement between the first and second reviewers.

Assessment of the Methodological Quality of the Included Studies

The quality assessment was performed by modifying the risk of bias methodology used in previous systematic reviews for in-vitro studies [18,19]. Two independent reviewers assessed the following parameters: (1) sample size calculation, (2) teeth randomization, (3) control group, (4) standardization of root canal anatomy (curvature), (5) bias due to operator variability (single operator), and (6) correct statistical analysis. “Yes” and “No” were assigned to the parameters reported or missing in the included studies, respectively. The low risk of bias was assigned when five to six parameters reported “Yes,” when three to four parameters reported “Yes,” a moderate risk of bias was given, and a high risk of bias was assigned when only one or two items were reported. Any disagreements between the first and second reviewers were resolved through discussion to reach a consensus and/or by a third reviewer (KS).

Meta-Analysis

Quantitative data synthesis was carried out using the software program RevMan Software 5.4 (Cochrane Collaboration, Copenhagen, Denmark). Instrumentation time and canal transportation was selected as the outcome.

Statistical heterogeneity between studies was analyzed using the I2 value, with low, medium, and high heterogeneity indicated by values of 25%, 50%, and greater than 50%, respectively [20]. If the I2 score was up to 50%, a fixed-effects model was used, whereas a random-effects model was applied if the I2 score was above 50%.

Results

Study Selection

**Figure 1:**
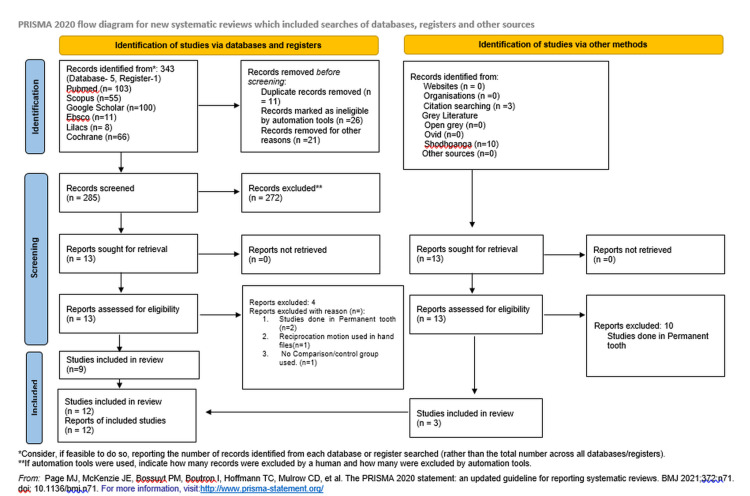
PRISMA flow diagram. PRISMA: Preferred Reporting Items for Systematic Reviews and Meta-Analyses

The details of the literature searches performed are provided in the PRISMA flowchart (Figure 1).

A total of 353 articles were identified from five databases, one register, and other methods. Title and abstract screening of the articles resulted in the exclusion of 272 articles. The full text of the remaining 26 articles was retrieved. After full-text assessment and application of inclusion and exclusion criteria, 12 articles were included in the qualitative analysis. The quantitative analysis was performed for five articles for instrumentation time and two articles for transportation due to a lack of data in the other articles. The reasons for exclusion [21-24] are presented in Table 1.

**Table 1:** Excluded studies.

Studies	Reasons for exclusion
Burklein et al. [21], Kishore et al. [22]	Studies done on permanent tooth
Jeevanandan et al. [23]	Reciprocation motion used in hand files
Pinheiro et al. [24]	No comparison/control group used

Study Characteristics and Quality Assessment

The characteristics of the 12 included studies [7,25-35] are described in Table 2.

**Table 2:** Characteristics of the studies included in this systematic review.

Authors	Year	Country	Number of samples	Tooth type	Groups	Canal curvature	Methodology	Main results of the study	Other important findings
Katge et al. [25]	2014	India	120 root canals (30 per group)	Maxillary and mandibular primary molars	Stainless steel K files (SS), protaper (PT), wave one, (WO) control group (CG)	Moderate root angulation	Evaluation of cleaning efficacy - Indian ink removal. Examined under stereomicroscope. Instrumentation time was recorded	WO was better than the PT and K-file regarding cleaning efficacy and instrumentation time	SS showed less ink removal due to poor cutting efficacies
Kucukyilmaz et al. [26]	2015	Turkey	45 (15 per group)	Single rooted primary canine teeth	PT, R25 Reciproc file (RP), OneShape file (OS)	<5°	Apically extruded debris and irrigant were evaluated by the method of Myers and Montgomery. Instrumentation time was recorded	Apically extruded debris RP (0.000378 ± 0.000271 gr) < OS (0.000558 ± 0.000171 gr). Apically extruded irrigant more in RP (1.121666). Instrumentation time RP	All instrumentation systems produced apical extrusion of debris and irrigation solution
Prabhakar et al. [7]	2016	India	24	Human primary teeth (16 molars, 6 incisors, 2 canines)	WO, OS	Not reported (NR)	Cone-beam computed tomography (CBCT) to assess canal transportation, canal centering ability, and dentin thickness (coronal, middle and apical level). Instrumentation time was recorded	Dentin thickness- WO=OS Canal transportation- WO	WO proved to be a faster and safer system with fewer procedural errors
Ramazani et al. [27]	2016	Iran	64	Mesiobuccal canals of primary mandibular second molars	CG, SS, M-two, RP	20-40°	Cleaning efficacy - India ink removal - cleared teeth were examined under a stereomicroscope. Shaping ability – CBCT. Instrumentation time was measured using a digital chronometer and examined under microscopic magnification for file fracture	RP showed better mean rank of cleaning efficacy. Shaping ability – Good. Taper - Mtwo>RP>K file. Less instrumentation time - RP	RP - suitable file for canal preparation of primary teeth
Pathak [28]	2016	India	84	30 root canals for each group	SS, Mtwo, WO, CG	Moderate root angulation	Cleaning efficacy - India Ink. Cleared teeth were examined under a stereomicroscope. Instrumentation time was recorded by a chronometer	WO better than Mtwo and SS. Instrumentation time WO	
Gungor and Kustarci [29]	2016	Turkey	60	Mesiobucccal canals of primary maxillary second molar teeth	Two NiTi file systems [Twisted File Adaptive (TFA) and Reciproc (RP)] and two irrigation techniques [Conventional needle irrigation (CNI) and laser-activated irrigation (LAI)]	Moderate root curvature (angles of curvature ranging between 10° and 20°)	Debris extrusion - Myers and Montgomery method	CNI groups extruded less debris than LAI groups with both TFA and RP systems	All instrumentation and irrigation techniques caused debris extrusion
Nazari Moghaddam et al. [30]	2017	Iran	28	96 canals of primary maxillary and mandibular molars	RP Mtwo, CG	NR	Cleaning efficacy - India Ink. Cleared teeth were examined under a stereomicroscope. Instrumentation time was recorded	Coronal and middle thirds of the roots, RP showed better cleanliness than Mtwo. Instrumentation time RP	
Arslan et al. [31]	2019	Turkey	75	Distal canals of primary mandibular molar teeth	SS, PT, Twisted File (TF), OS, and RP	Moderate root angulation	Debris and smear layer removal – sputter-coated with gold and prepared for scanning electron microscope (SEM). Instrumentation time was recorded	Instrumentation time - RP and OS - significantly less preparation time than all other groups (p < 0.001). Residual and smear removal - PT had significantly better results than the OS. RP had worse cleaning efficacy results than PT (p < 0.05)	
Selivany et al. [32]	2019	Iraq	60	Lower primary second molar	SS, OS , Wave One-Gold (WOG)	NR	Canal transportation, centring ability, dentin thickness - CBCT. Instrumentation time - digital chronometer	Canal transportation, centering ability, dentin thickness, instrumentation time - no significant difference between OS and WOG. Significant difference with SS	Single file system - reduction of instrumentation time and maintenance of original shape of the root canal
Fonseca et al. [33]	2020	Brazil	24	48 canals	Protaper Next (PN), WOG, Protaper Universal (PU), negative control	Moderate root angulation (10–20°)	Microbiological assessment for Enterococcus faecalis ATCC 29212-colony-forming units per millimeter (CFU/mL) was counted	No significant difference in microbial reduction between the PN, WOG, and PU	Smaller tapering in PN and WOG - favorable to preserve dentine
Barasuol et al. [34]	2021	Brazil	90	Root canals of primary molars	SS, ProDesign Logic (PDL), and RP	10–20° and radii >5 mm	Canal transportation, centring ability, percentage of dentin removal , perforations - micro CT evaluation	Perforations - 2 (RP), 1 (PDL), 0 (SS) canal transportation (middle third) RP>SS> PDL. Mean instrumentation time RP	
Yüksel et al. [35]	2021	Turkey	30	Mesial canals of mandibular second molars	OS, XP-endo Shaper, (XP-ES), (WOG)		Microcrack formation and shaping of danger zone - Micro CT	Dentin reduction in the danger zone area. OS < XP-ES	Rotary systems were superior to reciprocating in terms of shaping ability in the danger zone

The sample size for each group in the reported studies varied from 15 to 30 root canals per group with an average curvature ranging between 5° and 40°. The studies were performed on the anterior and posterior teeth of the maxilla and mandible. The quality assessment of the studies is summarized in Table 3.

**Table 3:** Risk of bias assessment for the studies included in this systematic review.

Study	Sample size calculation	Teeth randomization	Control group	Standardization of root canal anatomy (curvature); value should be given	Was the preparation done by a single operator?	Correct statistical analysis (mean and SD)	Overall bias
Katge et al. [25]	No	Yes	Yes	No	Yes	Yes	Moderate risk
Kucukyilmaz et al. [26]	No	No	No	Yes	Yes	Yes	Moderate risk
Prabhakar et al. [7]	No	No	No	No	No	Yes	High risk
Ramazani et al. [27]	Yes	Yes	Yes	Yes	Yes	Yes	Low risk
Pathak [28]	No	Yes	Yes	No	Yes	Yes	Moderate risk
Gungor and Kustarci [29]	No	Yes	No	Yes	Yes	Yes	Moderate risk
Nazari Moghaddam et al. [30]	No	Yes	Yes	No	No	No	High risk
Arslan et al. [31]	No	No	No	Yes	Yes	Yes	Moderate risk
Selivany et al. [32]	No	Yes	Yes	No	Yes	Yes	Moderate risk
Fonseca et al. [33]	Yes	Yes	Yes	Yes	No	No	Moderate risk
Barasuol et al. [34]	Yes	Yes	Yes	Yes	Yes	Yes	Low risk
Yüksel et al. [35]	No	Yes	No	Yes	Yes	No	Moderate risk

**Figure 2:**
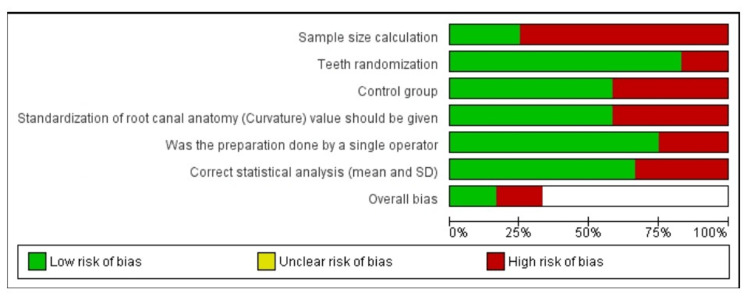
The risk of bias graph.

**Figure 3:**
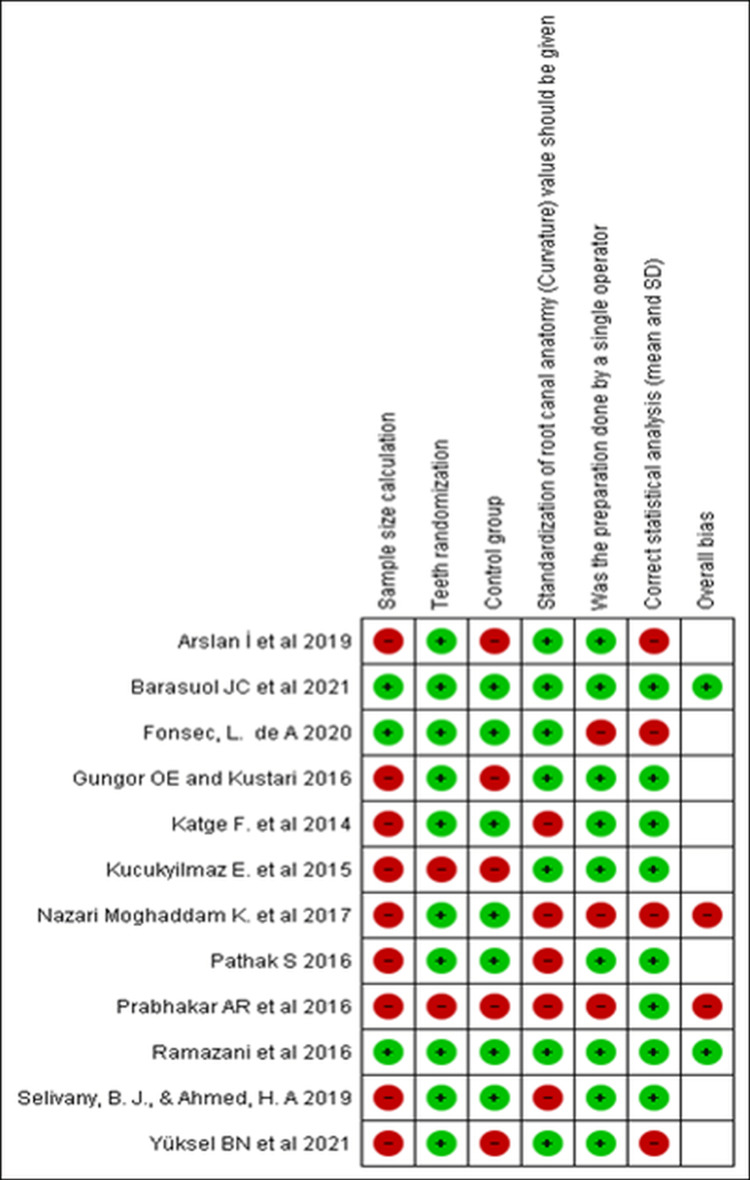
Risk of bias summary.

Out of the 12 included studies, two were assessed as high risk, eight with moderate risk, and two with low risk (Figures 2, 3).

Meta-Analysis

**Figure 4:**
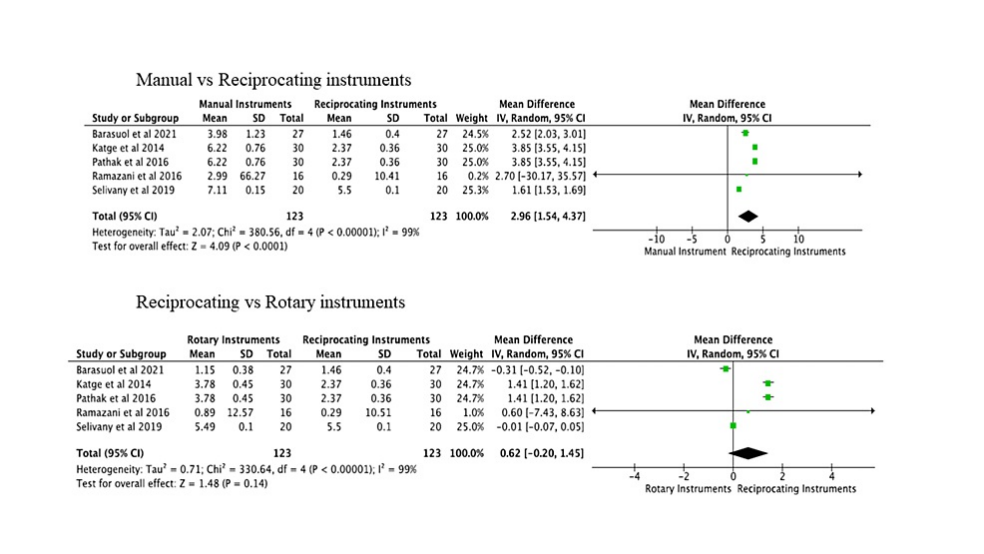
Forest plot for instrumentation time.

Of the 12 included studies, five presented relevant data on the outcome instrumentation time and two on canal transportation. The meta-analysis for instrumentation time between reciprocating and manual instruments was significant with pooled odds ratio (2.96,1.54, 4.37) at 95% confidence interval with substantial heterogeneity. There was no statistical significance when comparing reciprocating and rotary instruments with pooled odds ratio (0.63, -0.19, 1.45) at 95% confidence interval. The results are shown as a forest plot in Figure 4.

**Figure 5:**
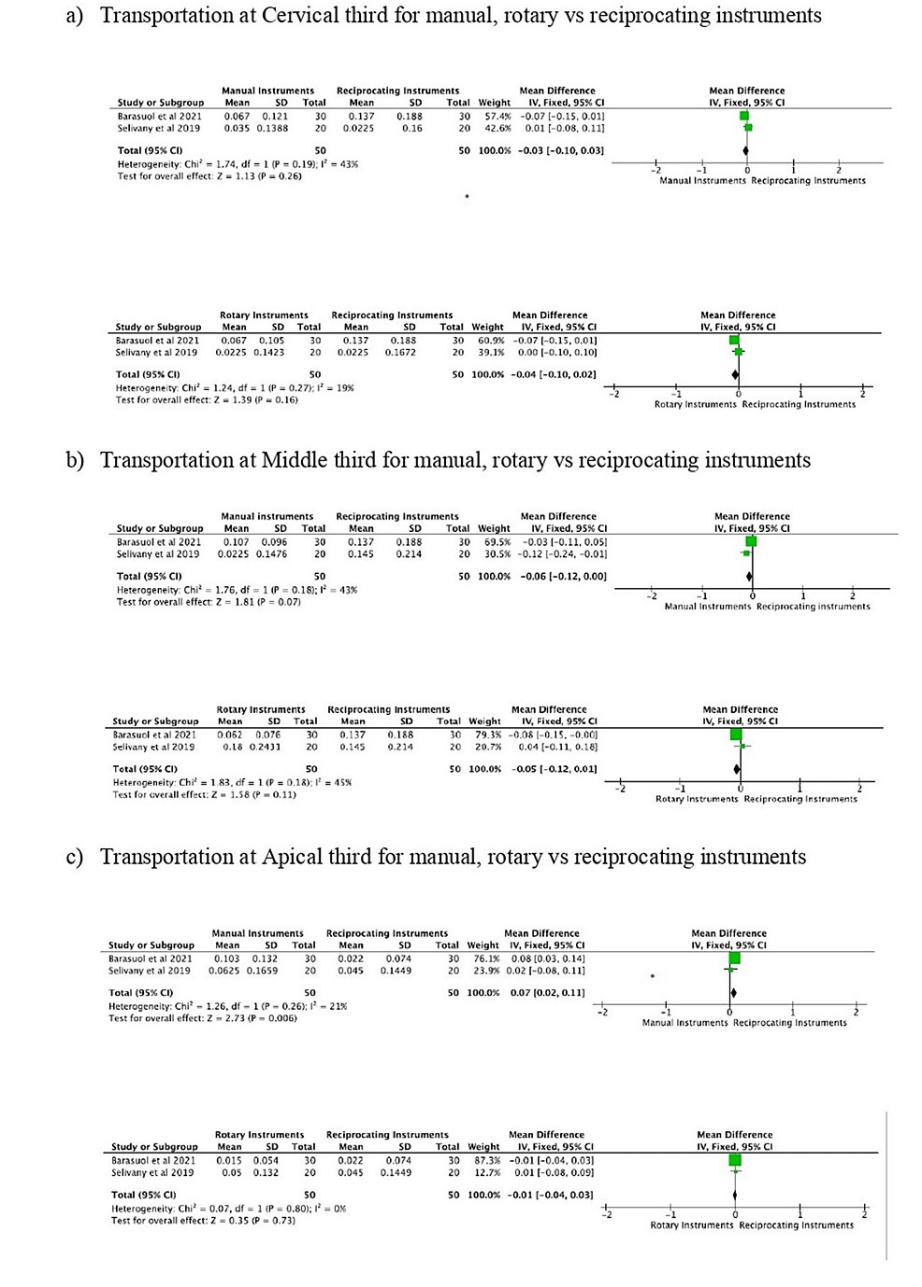
Forest plot for canal transportation.

The quantitative synthesis for canal transportation at the cervical, middle, and apical levels showed no significant difference between reciprocating and rotary instruments. There was no significant difference between reciprocating and hand instruments. The results are shown as a forest plot in Figure 5.

Properties and Outcomes Assessed

The canal transportation, centering ability, dentin thickness/canal volume and surface area, apically extruded debris, residual debris and smear layer, cleaning efficacy, microcrack formation, instrumentation time, instrument failure, and shaping ability were assessed in this systematic review. The details of the outcomes assessed are presented in Table 4.

**Table 4:** Outcomes assessed in the included studies.

Centering ability	Transportation	Dentin thickness/canal volume and surface area	Instrumentation time	Apically extruded debris	Cleaning efficacy	Micro crack formation	Instrument failure	Shaping ability	Disinfection of the root canal	Residual debris and smear layer
Prabhakar et al. [7]	Prabhakar et al. [7]	Prabhakar et al. [7]	Prabhakar et al. [7]	Kucukyilmaz et al. [26]	Katge et al. [25]	Yüksel et al. [35]	Ramazani et al. [27]	Ramazani et al. [27]	Fonseca et al. [33]	Arslan et al. [31]
Selivany et al. [32]	Selivany et al. [32]	Selivany et al. [32]	Katge et al. [25]	Gungor and Kustarci [29]	Ramazani et al. [27]			Yüksel et al. [35]		
	Barasuol et al. [34]	Barasuol et al. [34]	Kucukyilmaz et al. [26]		Pathak [28]					
			Ramazani et al. [27]		Nazari Moghaddam et al. [30]					
			Pathak [28]							
			Nazari Moghaddam et al. [30]							
			Arslan et al. [31]							
			Selivany et al. [32]							
			Barasuol et al. [34]							

Discussion

The success of the pulpectomy depends on the complete cleaning and shaping of root canals in primary teeth [15]. However, the presence of accessory and lateral canals, fins, inosculates between canals, and thin roots in primary teeth makes anatomy more complicated than permanent teeth and difficult to clean [36]. The introduction of engine-driven NiTi instruments in pediatric dentistry has resulted in funnel-shaped canal preparation in less time [4]. The reciprocating instruments have the advantage of reducing the torsional and flexural stresses as these files exert balanced force on root canal instrumentation [37].

Canal transportation is defined as the removal of canal wall structure on the outside curve in the apical half of the canal. This is due to the tendency of files to return to their original linear shape during canal preparation [38]. Reciproc instruments with a 0.08 mm taper showed better canal transportation than Prodesign Logic instruments [34]. Reciprocating and rotary instruments showed less canal transportation when compared with hand instruments [7,32]. From the available literature, it was observed that canal transportation was commonly seen in the middle third for both rotary and reciprocating instruments.

The centering ability is defined as the ability of the instrument to remain centered in the root canal with symmetrical preparation of the root canal along with equal removal of dentin from the root canal [39]. The reciprocating and rotary instruments showed better canal centering ability than hand K instruments [7,32]. This could be due to the better flexibility of the NiTi instruments and programmed torque and speed control.

The removal of dentin thickness was assessed on the axial section of the outer surface of the tooth to the periphery of the pulp space. This was done at the cervical, middle, and apical levels [40]. The hand K instruments removed more dentin compared with reciprocating and rotary instruments [7,32,34]. Dentin was commonly removed at the apical level [32].

Instrumentation time is defined as the time taken to complete the entire cleaning and shaping procedure of a tooth. Numerous studies have evaluated the difference in the instrumentation time using reciprocation versus rotary file systems, with most reporting that less time is needed for reciprocation than rotary instruments [7,25-28,30,31], while three studies reported no difference [29,32,34]. Meta-analysis performed in this study also favors reciprocating versus rotary filing, one of the major reasons being the fewer files used in the reciprocating file system when compared to the rotary file system; however, the results were not statistically significant. In addition, due to the counterclockwise rotation of the reciprocation file system, the removal of dentin and shaping of the canals is much faster. From the studies, it can be concluded that rotary and reciprocation instruments showed much lower instrumentation time than hand instruments.

The extrusion of debris and irrigants during cleaning and shaping of the root canal preparation results in severe inflammation reaction [40]. The factors for debris extrusion are apical morphology, size of needle syringe, and technique of root canal preparation [41]. Kucukyilmaz et al. reported that the lowest amount of extruded debris was observed with the Reciproc than Protaper and the highest amount was observed with the OneShape instruments [26]. In another study, it was found that all instrumentation and irrigation techniques caused debris extrusion. However, no significant differences were found between the instrumentation and irrigation techniques used [29].

The success of endodontic treatment depends on the efficient cleaning of the canals [27]. Reciprocating instruments showed superior canal cleanliness than rotary instruments [25-28,30].

The distal wall of the mesial root and the mesial wall of the distal root at the furcal region are considered danger zones of primary mandibular molars. The more dentin is removed from this region, the more microcracks are formed [42]. The microcracks were observed in the middle third rather than in the coronal and apical regions in both rotary and reciprocating instruments [35].

When an instrument maintains a gradual narrowing of the canal from the coronal to the apical third of the canal, it has excellent shaping ability. The rotary and reciprocating instruments showed better shaping ability than hand instruments [27]. Yüksel et al. concluded that rotary systems were superior to reciprocating systems regarding shaping ability in the danger zone [35].

## Conclusions

Our study suggests that reciprocating instruments showed less instrumentation time to clean the root canals of primary teeth than hand instruments. However, there is no scientific evidence that reciprocating instruments require less instrumentation time when compared to rotary instruments. Regarding canal transportation, the reciprocating and rotary instruments showed less canal transportation than hand instruments but were not statistically significant. However, with regard to other parameters, there is no significant difference between rotary and reciprocating instruments. To assess the shaping ability of reciprocating instruments in primary teeth a well-designed randomized clinical trial in the future will enhance the clinical utilization of reciprocating instruments in pediatric dentistry.

## References

[1] Koshy S, Love RM (2004). Endodontic treatment in the primary dentition. Aust Endod J.

[2] Kuo CI, Wang YL, Chang HH, Huang GF (2006). Application of Ni‑Ti rotary files for pulpectomy in primary molars. J Dent Sci.

[3] Barr ES, Kleier DJ, Barr NV (1999). Use of nickel-titanium rotary files for root canal preparation in primary teeth. Pediatr Dent.

[4] Guelzow A, Stamm O, Martus P, Kielbassa AM (2005). Comparative study of six rotary nickel-titanium systems and hand instrumentation for root canal preparation. Int Endod J.

[5] Crespo S, Cortes O, Garcia C, Perez L (2008). Comparison between rotary and manual instrumentation in primary teeth. J Clin Pediatr Dent.

[6] Del Fabbro M, Afrashtehfar KI, Corbella S, El-Kabbaney A, Perondi I, Taschieri S (2018). In vivo and in vitro effectiveness of rotary nickel-titanium vs manual stainless steel instruments for root canal therapy: systematic review and meta-analysis. J Evid Based Dent Pract.

[7] Prabhakar AR, Yavagal C, Dixit K, Naik SV (2016). Reciprocating vs rotary instrumentation in pediatric endodontics: cone beam computed tomographic analysis of deciduous root canals using two single-file systems. Int J Clin Pediatr Dent.

[8] Shen Y, Cheung GS, Bian Z, Peng B (2006). Comparison of defects in ProFile and ProTaper systems after clinical use. J Endod.

[9] Paqué F, Zehnder M, De-Deus G (2011). Microtomography-based comparison of reciprocating single-file F2 ProTaper technique versus rotary full sequence. J Endod.

[10] Franco V, Fabiani C, Taschieri S, Malentacca A, Bortolin M, Del Fabbro M (2011). Investigation on the shaping ability of nickel-titanium files when used with a reciprocating motion. J Endod.

[11] Berutti E, Paolino DS, Chiandussi G, Alovisi M, Cantatore G, Castellucci A, Pasqualini D (2012). Root canal anatomy preservation of WaveOne reciprocating files with or without glide path. J Endod.

[12] Dietrich MA, Kirkpatrick TC, Yaccino JM (2012). In vitro canal and isthmus debris removal of the self-adjusting file, K3, and WaveOne files in the mesial root of human mandibular molars. J Endod.

[13] Robinson JP, Lumley PJ, Cooper PR, Grover LM, Walmsley AD (2013). Reciprocating root canal technique induces greater debris accumulation than a continuous rotary technique as assessed by 3-dimensional micro-computed tomography. J Endod.

[14] Kim HC, Kwak SW, Cheung GS, Ko DH, Chung SM, Lee W (2012). Cyclic fatigue and torsional resistance of two new nickel-titanium instruments used in reciprocation motion: Reciproc versus WaveOne. J Endod.

[15] Wycoff RC, Berzins DW (2012). An in vitro comparison of torsional stress properties of three different rotary nickel-titanium files with a similar cross-sectional design. J Endod.

[16] Page MJ, McKenzie JE, Bossuyt PM (2021). The PRISMA 2020 statement: an updated guideline for reporting systematic reviews. BMJ.

[17] Ouzzani M, Hammady H, Fedorowicz Z, Elmagarmid A (2016). Rayyan-a web and mobile app for systematic reviews. Syst Rev.

[18] Küçükkaya Eren S, Uzunoğlu-Özyürek E, Karahan S (2021). Influence of reciprocating and rotary instrumentation on microbial reduction: a systematic review and meta-analysis of in vitro studies. Restor Dent Endod.

[19] Silva EJ, Rover G, Belladonna FG, De-Deus G, da Silveira Teixeira C, da Silva Fidalgo TK (2018). Impact of contracted endodontic cavities on fracture resistance of endodontically treated teeth: a systematic review of in vitro studies. Clin Oral Investig.

[20] Higgins JP, Thompson SG, Deeks JJ, Altman DG (2003). Measuring inconsistency in meta-analyses. BMJ.

[21] Bürklein S, Hinschitza K, Dammaschke T, Schäfer E (2012). Shaping ability and cleaning effectiveness of two single-file systems in severely curved root canals of extracted teeth: Reciproc and WaveOne versus Mtwo and ProTaper. Int Endod J.

[22] Kishore A, Gurtu A, Bansal R, Singhal A, Mohan S, Mehrotra A (2017). Comparison of canal transportation and centering ability of Twisted Files, HyFlex controlled memory, and Wave One using computed tomography scan: an in vitro study. J Conserv Dent.

[23] Jeevanandan G, Thomas E (2018). Volumetric analysis of hand, reciprocating and rotary instrumentation techniques in primary molars using spiral computed tomography: an in vitro comparative study. Eur J Dent.

[24] Pinheiro SL, Ota CM, Romitti FM (2016). Morphological assessment and cleaning capacity of a reciprocating system in root canals of deciduous teeth.Brazilian. Braz Res Pediatr Dentistry Integrated Clin.

[25] Katge F, Patil D, Poojari M, Pimpale J, Shitoot A, Rusawat B (2014). Comparison of instrumentation time and cleaning efficacy of manual instrumentation, rotary systems and reciprocating systems in primary teeth: an in vitro study. J Indian Soc Pedod Prev Dent.

[26] Kucukyilmaz E, Savas S, Saygili G, Uysal B (2015). Evaluation of apically extruded debris and irrigant produced by different nickel-titanium instrument systems in primary teeth. J Contemp Dent Pract.

[27] Ramazani N, Mohammadi A, Amirabadi F, Ramazani M, Ehsani F (2016). In vitro investigation of the cleaning efficacy, shaping ability, preparation time and file deformation of continuous rotary, reciprocating rotary and manual instrumentations in primary molars. J Dent Res Dent Clin Dent Prospects.

[28] Pathak S (2016). In vitro comparison of K-file, Mtwo, and WaveOne in cleaning efficacy and instrumentation time in primary molars. CHRISMED J Health Res.

[29] Gungor OE, Kustarci A (2016). Evaluation of apically extruded debris using two Niti systems associated with two irrigation techniques in primary teeth. J Clin Pediatr Dent.

[30] Nazari Moghaddam K, Farajian Zadeh H, Farajian Zadeh N (2017). Comparison of cleaning efficacy and instrumentation time of Reciproc and Mtwo rotary systems in primary molars. J Islam Dent Assoc Iran.

[31] Arslan İ, Aydınoglu S, Baygin Ö, Tuzuner Ö, Sirin M (2019). Comparative analysis of manual, rotary and reciprocal systems on primary teeth root canals: an in vitro scanning electron microscopy study. Cumhuriyet Dent J.

[32] Selivany BJ, Ahmed HA (2020). Analysis of canal transportation, centering ability and remaining dentin thickness of different single file rotary systems in primary teeth; a CBCT assessment. J Duhok Univ.

[33] Fonseca LA, Cangussu RA, Oliveira AS, Pinheiro LS, Shitsuka C, Duarte DA (2020). Comparison of endodontic disinfection of primary teeth root canals using rotary and reciprocating system: an in vitro study. Res Soc Dev.

[34] Barasuol JC, Alcalde MP, Bortoluzzi EA, Duarte MA, Cardoso M, Bolan M (2021). Shaping ability of hand, rotary and reciprocating files in primary teeth: a micro-CT study in vitro. Eur Arch Paediatr Dent.

[35] Yüksel BN, Öncü A, Çelİkten B, Bİlecenoğlu B, Orhan AI, Orhan K (2022). Micro-CT evaluation of 'danger zone' and microcrack formation in mesial root canals of primary teeth with single-file rotary and reciprocating systems. Int J Paediatr Dent.

[36] Kurthukoti AJ, Sharma P, Swamy DF, Shashidara R, Swamy EB (2015). Computed tomographic morphometry of the internal anatomy of mandibular second primary molars. Int J Clin Pediatr Dent.

[37] You SY, Bae KS, Baek SH, Kum KY, Shon WJ, Lee W (2010). Lifespan of one nickel-titanium rotary file with reciprocating motion in curved root canals. J Endod.

[38] American Association of Endodontists (2003). American Association of Endodontists Glossary of Endodontic Terms. Chicago: AAE.

[39] Kandaswamy D, Venkateshbabu N, Porkodi I, Pradeep G (2009). Canal-centering ability: an endodontic challenge. J Conserv Dent.

[40] Tanalp J, Kaptan F, Sert S, Kayahan B, Bayirl G (2006). Quantitative evaluation of the amount of apically extruded debris using 3 different rotary instrumentation systems. Oral Surg Oral Med Oral Pathol Oral Radiol Endod.

[41] Altundasar E, Nagas E, Uyanik O, Serper A (2011). Debris and irrigant extrusion potential of 2 rotary systems and irrigation needles. Oral Surg Oral Med Oral Pathol Oral Radiol Endod.

[42] Shantiaee Y, Dianat O, Paymanpour P, Nahvi G, Ketabi MA, Kolahi Ahari G (2015). Alterations of the danger zone after preparation of curved root canals using WaveOne with reverse rotation or reciprocation movements. Iran Endod J.

